# Extending the phenotype of *BMPER-*related skeletal dysplasias to ischiospinal dysostosis

**DOI:** 10.1186/s13023-015-0380-0

**Published:** 2016-01-04

**Authors:** Ekaterina Kuchinskaya, Giedre Grigelioniene, Anna Hammarsjö, Hye-Ran Lee, Lotta Högberg, Gintautas Grigelionis, Ok-Hwa Kim, Gen Nishimura, Tae-Joon Cho

**Affiliations:** Department of Clinical Pathology and Clinical Genetics, and Department of Clinical and Experimental Medicine, Linköping University, Linköping, Sweden; Department of Molecular Medicine and Surgery, Karolinska Institutet, Stockholm, Sweden; Department of Clinical Genetics, Karolinska University Hospital, Stockholm, Sweden; Division of Pediatric Orthopaedics, Seoul National University Children’s Hospital, Seoul, Republic of Korea; Department of Paediatrics and Department of Clinical and Experimental Medicine, Linköping University, Norrköping, Sweden; Department of Radiology, Woorisoa Children’s Hospital, Seoul, Republic of Korea; Department of Pediatric Imaging, Tokyo Metropolitan Children’s Medical Center, Tokyo, Japan

**Keywords:** Ischiospinal dysostosis, Diaphanospondylodysostosis, *BMPER*, Vertebral anomaly, Ischial hypoplasia

## Abstract

Ischiospinal dysostosis (ISD) is a polytopic dysostosis characterized by ischial hypoplasia, multiple segmental anomalies of the cervicothoracic spine, hypoplasia of the lumbrosacral spine and occasionally associated with nephroblastomatosis. ISD is similar to, but milder than the lethal/semilethal condition termed diaphanospondylodysostosis (DSD), which is associated with homozygous or compound heterozygous mutations of bone morphogenetic protein-binding endothelial regulator protein (*BMPER*) gene. Here we report for the first time biallelic *BMPER* mutations in two patients with ISD, neither of whom had renal abnormalities. Our data supports and further extends the phenotypic variability of *BMPER-*related skeletal disorders.

**Electronic supplementary material:**

The online version of this article (doi:10.1186/s13023-015-0380-0) contains supplementary material, which is available to authorized users.

Ischiospinal dysostosis (ISD) is a polytopic dysostosis characterized by minor facial dysmorphism, ischial hypoplasia and short stature with a short spine caused by vertebral anomalies including hypoplasia of the lumbosacral spine, scoliosis and segmental defects of the cervicothoracic spine [1]. Eight patients have been reported so far [2–4] (Additional file 1: Table S1). Some of them showed mono- or polycystic kidneys with or without nephroblastomatosis [3, 4], neurogenic bladder and neurological deficits of the lower extremities [2]. Parental consanguinity in one patient suggested autosomal recessive inheritance, but the causative gene has been *hitherto* unknown [2]. Diaphanospondylodysostosis (DSD) is a lethal/semilethal skeletal dysplasia, the phenotype of which is similar to, but more severe than, that of ISD [5–12] (Additional file 1: Tables S1 and Additional file 2: Table S2). Funari et al. identified mutations in the *BMPER* in four patients with DSD [13], and since then two additional DSD patients and three siblings with so-called attenuated form of DSD with *BMPER* mutations have been published [9, 12] (Table 1, Fig. 1). We report two ISD patients with biallelic mutations (three novel variants) in the *BMPER* gene, extending the spectrum of *BMPER*-related skeletal disorders.

**Table 1:** List of mutations of *BMPER* reported previously and in the current study

Number^a^	cDNA	Protein	Frequency in ExAC	In-silico analysis^b^	Zygosity in proband	Diagnosis	Reference and comment
1	c.925C>T	p.Gln309*	NR	NS	Homozygous	DSD	[13]
2	c.26_35del10ins14	p.Ala9Glufs*4	NR	NS	Compound heterozygous	DSD	[8, 10, 11, 13]; same patient in four articles
3	c.1032+5G>A		NR	Splice			
4	c.514C>T	p.Gln172*	NR	NS	Heterozygous^c^	DSD	[13]
5	c.1109C>T	p.Pro370Leu	NR	Deleterious	Compound heterozygous	DSD	[13]
6	c.1638T>A	p.Cys546*	NR	NS			
7	c.310C>T	p.Gln104*	NR	NS	Homozygous	DSD	[9]; two patients
8	c.251G>T	p.Cys84Phe	NR	Deleterious	Compound heterozygous	attenuated DSD^d^	[12]
9	c.1078+5G>A		NR	Splice			
10	c.416C>G	p.Thr139Arg	NR	Deleterious	Compound heterozygous	ISD	Current study
11	c.942G>A	p.Trp314*	NR	NS			
12	c.1672C>T	p.Arg558*	0.00004119^e^	NS	Homozygous	ISD	Current study
13^f^	c.1988G>A	p.Cys663Tyr	0.0000082	Deleterious	Heterozygous		Current study

*NR* not reported; *NS* nonsense mutation; *DSD* diaphanospondylodysostosis; *ISD* ischiospinal dysostosis

^a^ Mutation number is also used in Fig. 1

^b^ Prediction according to SIFT (http://sift-dna.org), AlignGVGD (http://agvgd.iarc.fr/), MutationTaster (http://www.mutationtaster.org/), ExAC (http://exac.broadinstitute.org) and PolyPhen-2 (http://genetics.bwh.harvard.edu/pph2)

^c^ Only one mutation identified in this patient

^d^ It was described as attenuated DSD in the reference, but we consider them more likely to be ISD

^e^ Not present in 2,040 normal alleles from a Korean population (in-house data)

^f^ Variant of unknown clinical significance, found in patient with mutation No. 12

**Fig. 1:**
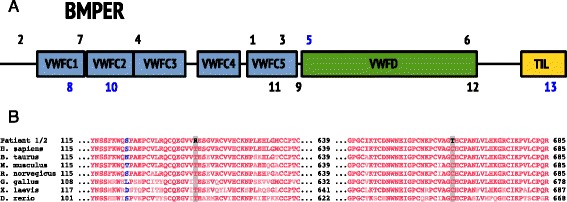
**a**: Locations of the mutations in the BMPER protein, previously reported (1–7) and identified in this study (8–11). Truncating mutations in black; missense in blue. **b**: Evolutionary comparison of the amino acids affected by novel missense mutations in this study. Blue and red colours indicate weakly and strongly conserved amino acids respectively

## Patients

Patient 1 was a 2-year-old girl, a second child born to a healthy non-consanguineous Swedish couple after an uncomplicated pregnancy. The older brother was healthy. The patient was born in week 41+0 after an uncomplicated delivery with BW 2590 g (*z* = −1.9), BL 46 cm (*z* = −1.9), OFC 33 cm (*z* =−1.0). The neonatal period and psychomotor development were unremarkable. At age 14 months, she had short stature with short trunk, hypoplastic thorax, protruding abdomen, and mild facial dysmorphism. Extremities were normal. At age two years her height was 76.2 cm (*z* = −3.3), she had hearing loss, mechanism of which is still under investigation, delayed speech development, and wheezing upon cold exposure and physical exercise. Repeated renal ultrasonography did not show nephrogenic rests or cysts, but will continue until age 7 years.

**Fig. 2:**
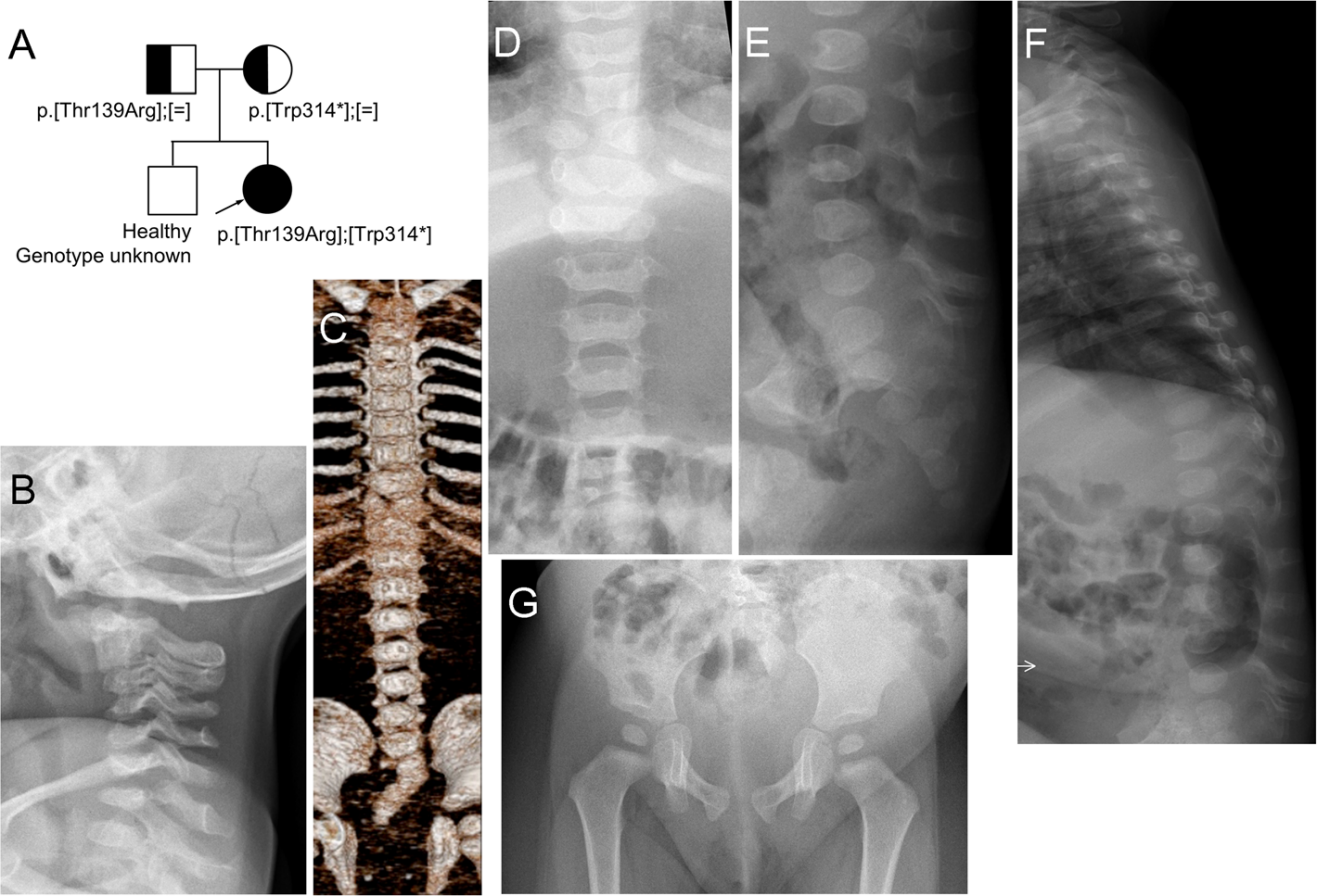
**a**: Pedigree and mutations in patient 1. **b**: Radiological examination at age 15 months shows narrowing of the intervertebral spaces of C2/3 and C3/4 presumably due to nonosseous synostosis. **c**-**f**: Radiological examinations at age 11 months. Butterfly vertebra is evident in T9. Only ten pairs of the ribs and 11^th^ rib of the right are seen. The lumbar vertebral bodies are small, and ossification of the neural arches is defective. Caudal narrowing of the lumbar interpedicular distance is seen. The sacrum is hypoplastic and deviated rightward. **g**: Ossification of the ischial rami is defective, and the ischiopubic synchondroses are wide

**Fig. 3:**
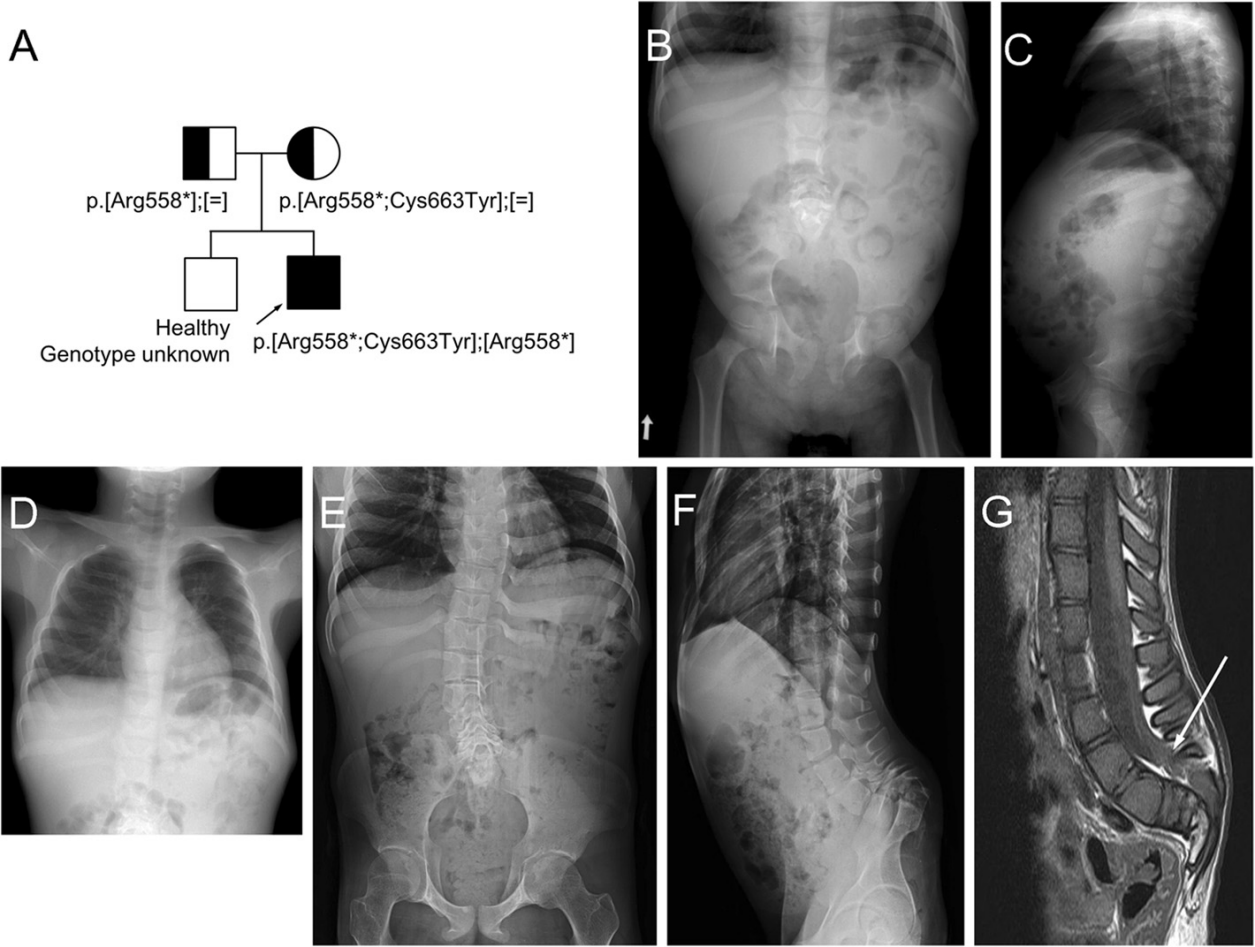
**a**: Pedigree and mutation in patient 2. **b**, **c**, **d**: Radiographs obtained at age 6 years show hypoplastic lumbar vertebral bodies, narrow interpedicular distance through the lumbar spine due to hypoplastic pedicles. Note absent coccygeal bones on the lateral spine, flared iliac wings, flat acetabulum and mild coxa valga. Short and tapering appearance of the bilateral ischial bones and wide ischiopubic junction is noted. Chest radiograph shows dysplastic and partly unossifed right upper ribs. **e**, **f**, **g**: Radiographs and MR imaging of the lumbosacral spine taken at age 19 years show mild thoracic and lumbar scoliosis with vertebral rotation, remarkable narrowing of the spinal canal with touching of the vertebral bodies to the posterior neural arch owing to absent/hypoplastic pedicles through lumbosacral vertebrae, and reveal lower lying spinal cord tethered to the lumbosacral junction (arrow). Pelvis AP radiograph shows hypoplastic ischium and inferior pubic rami, resulting in persistent gap between ischiopubic junction. Hip joint space is narrow

Patient 2 was a 19-year-old male, a second child of a healthy non-consanguineous Korean couple. He was born after an uncomplicated full-term pregnancy with BW 3190 g (z = −0.43), BL 46 cm (z = −1.42), and OFC 35 cm (z = 0.20). Respiratory distress immediately after birth required oxygen therapy. Abdominal distension, hydronephrosis and urethral stricture were noted during the neonatal period, but neither nephrogenic rests nor cysts. He showed mild facial dysmorphic features, a short trunk and pectus carinatum. At age three months, he required brief mechanical ventilation due to pneumonia and suffered from seasonal asthma attacks thereafter. Lack of urination control required intermittent catheterization from two years of age. He suffered from fecal incontinence and impaction. He stood with assistance and spoke only single words at age two years. Progressive right pes equinus deformity developed along with deterioration of lower extremity motor function, which limited outdoor ambulation to about five minutes at age 19 years. Untethering of the spical cord was considered but the patient was not compliant. His height was 143 cm (z = −5.67) and weight 27.6 kg (z = −9.23), and his school performance is normal. The radiographic phenotypes are summarized in Figs. 2 and 3 and clinical characteristics in Additional file 1: Table S1.

For patient 1, whole exome, subsequent Sanger sequencing and segregation analysis of the family revealed compound heterozygous mutations in the *BMPER*: NM_133468_4:c.[416C>G];[942G>A], p.[Thr139Arg];[Trp314*] (Fig. 3a). The amino acid substituted by the missense mutation is highly conserved among species (Fig. 3b). For patient 2, Sanger sequencing of the *BMPER* showed three sequence variants: NM_133468_4: c.[1672C>T;1988G>A];[c.1672C>T], p.[Arg588*;Cys662Tyr];[Arg588*]. The two variants on the same allele were inherited from the mother and the single mutation from the father (Fig. 3a). The variant NM_133468.4:c.1988G>A; p.Cys663Tyr is rare and predicted to be pathogenic (Table 1), but as the truncating mutation p.Arg558* is located upstream, p.Cys663Tyr substitution is most probably not involved in pathogenesis of this patient’s ISD.

## Discussion

Previous reports ascertained biallelic *BMPER* mutations in six patients with DSD [9, 13] and three siblings with attenuated DSD [12]. In this report we show that ISD a disorder phenotypically similar, but much milder, than DSD is also caused by biallelic mutations in the *BMPER* gene.

The *BMPER* encodes a 658 amino acid protein, which regulates organogenesis through the BMP signaling pathway. It is highly expressed in lungs, brain and chondrocytes. The knockout mice for the Bmp-binding protein crossveinless 2 (Bmper) show defects of vertebral and cartilage development, renal hypoplasia, as well as abnormal lung alveoli [14]. The knockout mouse phenotype is recapitulated by the manifestations in patients with *BMPER* mutations. The most prominent features are vertebral segmentation anomalies and kidney abnormalities [2–4, 7–9, 11]. Kidney abnormalities (cysts and nephroblastomatosis) have been reported in most patients with DSD [7–11], but only in some patients with ISD [3, 4]. Patient 2 has hydronephrosis due to neurogenic bladder. However, neither renal cysts nor nephroblastomatosis were present in our patients. Our observation indicates that kidney abnormalities are not necessarily a feature in mildly affected patients with *BMPER* mutations.

Patient 1 is compound heterozygous for a missense variant p.Thr139Arg and for a nonsense mutation p.Trp314*, which could explain her milder phenotype. Patient 2 is homozygous for the nonsense mutation p.Arg558*, which is predicted to result in a stop codon in the von-Willebrand factor D domain and loss of Trypsin-inhibitor like domain (Fig. 1a). With the available data, it is impossible to predict which mutations cause DSD and which cause ISD. It may be that some nonsense mutations undergo nonsense-mediated mRNA decay, while others do not and that the latter may retain some residual function. Also, other genetic factors might modify the clinical phenotype. Further molecular studies are needed to elucidate the molecular impact of the different mutations in BMPER.

In conclusion, our report extends the phenotypic variabilities in BMPER-related skeletal disorders. This dysostosis family encompasses phenotypes from mild ISD to lethal DSD.

### Consent statement

The cases are reported with informed parental and patients consents and with permission from the regional Ethical Boards of the Karolinska University Hospital, Stockholm, Sweden and Seoul National University Hospital, Seoul, Korea.

## Additional files

Additional file 1: Table S1. Summary of the patients with ISD and DSD, previously reported and in the current study. (DOCX 31 kb) Additional file 2: Table S2. Fetuses reported in the literature suspected to have DSD (DOCX 23 kb)

## Abbreviations

ISD: Ischiospinal dysostosis
DSD: Diaphanospondylodysostosis
BMPER: Bone morphogenetic protein-binding endothelial regulator protein
BW: Birth weight
BL: Birth length
OFC: Occipitofrontal circumference
VWFC: Von Willebrand factor type C domain
VWFD: Von Willebrand factor type D domain
TIL: Trypsin inhibitor-like domain
