# Supplementary material for: Extending the phenotype of BMPER-related skeletal dysplasias to ischiospinal dysostosis
Source: Orphanet J Rare Dis. 2016 Jan 4;11:1. doi: 10.1186/s13023-015-0380-0 (PMC4700746; doi:10.1186/s13023-015-0380-0)
Supplement: Additional file 1: Table S1. — Summary of the patients with ISD and DSD, previously reported and in the current study. (DOCX 31 kb) [file 13023_2015_380_MOESM1_ESM.docx]

**Additional Table 1.** Summary of the patients with ISD and DSD, previously reported and in the current study

| **Patient number** | **Clinical diagnosis** | **Gender** | **Survival^1^** | **Consanguinity** | **Ethnicity** | **Height^2^**  (z score) | **Short trunk** | **Kidney pathology** |
| --- | --- | --- | --- | --- | --- | --- | --- | --- |
| 1 | ISD | Female | > 33yr | No | Japanese | -8.5 | Yes | NoM |
| 2 | ISD | Male | > 7yr | No | Japanese | -2.9 | Yes | NoM |
| 3 | ISD | Female | > 38yr | 1st cousins | Japanese | -5.6 | Yes | NoM |
| 4 | ISD | Male | > 3mo | No | Japanese | -5.7 | Yes | NoM |
| 5 | ISD | Male | > 6mo | No | Japanese | -7.2 | No | NoM |
| 6 | ISD | Male | > 10mo | No | Caucasian | 50th% | Yes | Polycystic, NB |
| 7 | ISD | Male | > 2yr | No | Korean | -3.9 | Yes | Polycystic |
| 8 | ISD | Female | > 5yr 11mo | No | Japanese | -5.3 | NoM | Monocystic, Lt. |
| 9 | DSD | Male | Stillborn | Yes | Mali | (45cm) | Yes | Polycystic, NB |
| 10 | DSD | Female | Stillborn | Yes | Mali | (44cm) | Yes | Polycystic, NB |
| 11 | DSD | Male | Neonatal death | No | Not mentioned | NoM | Yes | NoM |
| 12 | DSD | Male | Neonatal death or stillborn | No | Not mentioned | (47cm) | NoM | NoM |
| 13 | DSD | Male | Neonatal death | No | Hispanic | NoM | Yes | Polycystic, NB |
| 14 | DSD | Male | Neonatal death | No | European | NoM | Yes | NoM |
| 15 | DSD | Female | Neonatal death | NoM | Caucasian | NoM | Yes | Polycystic |
| 16 | DSD | Male | 5yr (Wilms tumor) | No | European | NoM | Yes | Polycystic, Wilms tumor |
| 17 | DSD | Female | 15mo (Acute RSV) | 2nd cousins | Arabic | NoM | Yes | Polycystic |
| 18 | DSD | Female | 4mo (Resp.insufficiency) | No | Arabic | NoM | Yes | Normal |
| 19 | DSD^5^ | Male | > 13 yr | No | British-European | -4.7 | Yes^6^ | Normal |
| 20 | DSD^5^ | Male | > 6 ~ 13 yr | No | British-European | -3.8 | Yes^6^ | Normal |
| 21 | DSD^5^ | Male | > 6 yr | No | British-European | -5.0 | Yes^6^ | Normal |
| 22 | ISD | Female | > 2yr | No | Swedish | -3.3 | Yes | Normal |
| 23 | ISD | Male | > 19yr | No | Korean | -5.7 | Yes | Hydronephrosis |

**Additional Table 1. Continued**

| **Patient number** | **Respiratory distress at birth** | **Facial dysmorphism** | **Neurologic deficit** | **Other abnormalities** | **BMPER mutation^3^** | **Reference** |
| --- | --- | --- | --- | --- | --- | --- |
| 1 | None | None | Paraparesis, feet deformities, neurogenic bladder |  | NA | [2] |
| 2 | None | Yes | Paraparesis, feet deformities |  | NA | [2] |
| 3 | None | None | Paraparesis, feet deformities |  | NA | [2] |
| 4 | Yes, intubation | None | NoM |  | NA | [2] |
| 5 | None | None | NoM | Collodion baby, cryptorchidism, inguinal hernia, heart anomaly, alopecia | NA | [2] |
| 6 | None | Yes | None |  | NA | [3] |
| 7 | None | None | Developmental delay |  | NA | [4] |
| 8 | None | NoM | Developmental delay | Cleft palate | NA | [4] |
| 9 | - | NoM | Feet deformities |  | NA | [7] |
| 10 | - | NoM | NoM |  | NA | [7] |
| 11 | Yes, fatal | Yes | NoM |  | NA | [7] |
| 12 | NoM | NoM | NoM | Cleft palate | NA | [7] |
| 13 | Yes, fatal | Yes | NoM |  | NA | [8] |
| 14 | Yes, fatal | Yes | NoM | Inguinal hernia | NA | [8] |
| 15 | Yes, intubation | NoM | NoM | Tracheomalacia | NA | [8] |
| 16 | Yes, intubation | Yes | Peripheral neuropathy due to spinal cord anomaly | Hearing loss | 2 & 3 | [8,10,11,13] |
| 17 | Yes, intubation | Yes | None |  | 7 | [9] |
| 18 | Yes, NICU care | None | NoM | Nail hypoplasia | 7 | [9] |
| 19^4^ | None | NoM | NoM |  | 8 & 9 | [12] |
| 20^4^ | None | NoM | NoM |  | 8 & 9 | [12] |
| 21^4^ | None | NoM | NoM |  | 8 & 9 | [12] |
| 22 | None | Yes^7^ | None | Hearing loss | 10 & 11 | Current study |
| 23 | Yes, oxygen | Yes^8^ | Paraparesis, feet deformities, neurogenic bladder |  | 12 & 13 | Current study |

^1^ Inequality sign means the patient survived over the age mentioned. Age without inequality sign means the age when the patient died, followed by cause of death.

^2^ Height in z-value or centile at the latest follow-up (according to the corresponding population growth charts). The number in parentheses is birth length.

^3^ Refer to Table 1 for mutation numbers

^4^ Patients 19, 20, and 21 are siblings.

^5^ It was described as attenuated DSD in the reference, but we consider them more likely to be ISD.

^6^ Congenital kyphoscoliosis with multiple vertral and rib anomalies

^7^ Prominent forehead, short upturned nose, depressed nasal bridge, marked eyebrows

^8^ Slightly prominent forehead, marked eyebrows, depressed nasal bridge, prognathism, low set ears

ISD: ischiospinal dysostosis; DSD: diaphanospondylodysostosis; RSV: respiratory syncytial virus infection; BL: birth length; NoM: not mentioned; NB: nephroblastomatosis; NA: not available; Lt: left side
