# Supplementary material for: Extending the phenotype of BMPER-related skeletal dysplasias to ischiospinal dysostosis
Source: Orphanet J Rare Dis. 2016 Jan 4;11:1. doi: 10.1186/s13023-015-0380-0 (PMC4700746; doi:10.1186/s13023-015-0380-0)
Supplement: Additional file 2: Table S2. — Fetuses reported in the literature suspected to have DSD (DOCX 23 kb) [file 13023_2015_380_MOESM2_ESM.docx]

**Additional Table 2.** Fetuses reported in the literature suspected to have DSD

| **Patient**  **Number** | **Diagnosis** | **Gender** | **GA** | **Consanguinity** | **Ethnicity** | **Kidney pathology** | **Autopsy / Fetal ultrasonography** | **Radiography** | **Reference & Comment** |
| --- | --- | --- | --- | --- | --- | --- | --- | --- | --- |
| 1 | "a new syndrome" | Female | 23 | NoM | Afro-Caribbean | Polycystic | Short, bell-shaped chest, distended abdomen, omphalocele | 10 ribs, rib gaps, central clefting of vertebrae | [5], Dichorionic twins |
| 2 | "a new syndrome" | Female | 23 | NoM | Afro-Caribbean | Polycystic | Bell-shaped chest, distended abdomen | 9 ribs, rib gaps, central clefting of vertebrae |  |
| 3 | "severe malformation syndrome" | Female | 20 | None | Caucasian | Cystic, nephroblastomatosis | Thoracic hypoplasia, short neck, protruding abdomen, absent lumbosacral segment | Deficient ossification of vertebrae and ribs, rib gaps | [6], Siblings |
| 4 | "severe malformation syndrome" | Male | 17 | None | Caucasian | Polycystic | Thoracic hypoplasia, broad and shortened ribs, cartilaginous lumbosacral segment | Deficient vertebra and ischium ossification, rib gaps |  |
| 5 | "severe malformation syndrome" | Unknown | 12+6 | None | Caucasian | Cystic, nephroblastomatosis | Deficient ossification of thoracic and lumbar spine | Deficient vertebra ossification ribs, rib gaps |  |
| 6 | DSD | Female | 18 | None | Northern European | NoM | Short neck and trunk, low-set ear, small nose, hypertelorism, webbed neck | No ossification of T and L-spine, rib gaps | [8], Siblings of patient 10, Additional Table 1 |
| 7 | DSD | Male | 12 | None | Northern European | NoM | Incomplete development of spine and ribs, nuchal cystic hygroma, crab-like ribs | Not performed |  |
| 8 | DSD | Unknown | 13 | 2^nd^  cousin | Arabic | NoM | Nuchal translucency, lower spine not visualized | Not performed | [9], Siblings of patient 17, Additional Table 1 |
| 9 | DSD | Unknown | 13 |  | Arabic | NoM | Nuchal translucency, lower spine not visualized | Not performed |  |

GA: gestational age at termination in weeks; NoM: not mentioned; DSD: diaphanospondylodysostosis
